# Supplementary material for: End Sequence Analysis Toolkit (ESAT) expands the extractable information from single-cell RNA-seq data
Source: Genome Res. 2016 Oct;26(10):1397–410. doi: 10.1101/gr.207902.116 (PMC5052061; doi:10.1101/gr.207902.116)
Supplement: Supplemental Material [file supp_gr.207902.116_Supp_Materials.tar.gz › ESAT-esat_v0.1/doc/html/class_jama_1_1_singular_value_decomposition.html]

ESAT: Jama.SingularValueDecomposition Class Reference


ESAT


- Main Page
- Related Pages
- Packages
- Classes
- Files

- Class List
- Class Index
- Class Hierarchy
- Class Members

All Classes Namespaces Files Functions Variables Enumerator Pages

- Jama
- SingularValueDecomposition

Public Member Functions |
List of all members

Jama.SingularValueDecomposition Class Reference

Inheritance diagram for Jama.SingularValueDecomposition:

[legend]

Collaboration diagram for Jama.SingularValueDecomposition:

[legend]

|  |  |
| --- | --- |
| Public Member Functions | |
|  | SingularValueDecomposition (Matrix Arg) |
|  | |
| Matrix | getU () |
|  | |
| Matrix | getV () |
|  | |
| double[] | getSingularValues () |
|  | |
| Matrix | getS () |
|  | |
| double | norm2 () |
|  | |
| double | cond () |
|  | |
| int | rank () |
|  | |

## Detailed Description

Singular Value Decomposition.

For an m-by-n matrix A with m >= n, the singular value decomposition is an m-by-n orthogonal matrix U, an n-by-n diagonal matrix S, and an n-by-n orthogonal matrix V so that A = U\*S\*V'.

The singular values, sigma[k] = S[k][k], are ordered so that sigma[0] >= sigma[1] >= ... >= sigma[n-1].

The singular value decompostion always exists, so the constructor will never fail. The matrix condition number and the effective numerical rank can be computed from this decomposition.

## Constructor & Destructor Documentation

|  |  |  |  |  |  |
| --- | --- | --- | --- | --- | --- |
| Jama.SingularValueDecomposition.SingularValueDecomposition | ( | Matrix | *Arg* | ) |  |

Construct the singular value decomposition

Parameters
:   |  |  |
    | --- | --- |
    | A | Rectangular matrix |

Returns
:   Structure to access U, S and V.

## Member Function Documentation

|  |  |  |  |  |
| --- | --- | --- | --- | --- |
| double Jama.SingularValueDecomposition.cond | ( |  | ) |  |

Two norm condition number

Returns
:   max(S)/min(S)

|  |  |  |  |  |
| --- | --- | --- | --- | --- |
| Matrix Jama.SingularValueDecomposition.getS | ( |  | ) |  |

Return the diagonal matrix of singular values

Returns
:   S

|  |  |  |  |  |
| --- | --- | --- | --- | --- |
| double [] Jama.SingularValueDecomposition.getSingularValues | ( |  | ) |  |

Return the one-dimensional array of singular values

Returns
:   diagonal of S.

|  |  |  |  |  |
| --- | --- | --- | --- | --- |
| Matrix Jama.SingularValueDecomposition.getU | ( |  | ) |  |

Return the left singular vectors

Returns
:   U

|  |  |  |  |  |
| --- | --- | --- | --- | --- |
| Matrix Jama.SingularValueDecomposition.getV | ( |  | ) |  |

Return the right singular vectors

Returns
:   V

|  |  |  |  |  |
| --- | --- | --- | --- | --- |
| double Jama.SingularValueDecomposition.norm2 | ( |  | ) |  |

Two norm

Returns
:   max(S)

|  |  |  |  |  |
| --- | --- | --- | --- | --- |
| int Jama.SingularValueDecomposition.rank | ( |  | ) |  |

Effective numerical matrix rank

Returns
:   Number of nonnegligible singular values.

---

The documentation for this class was generated from the following file:

- java/Jama/SingularValueDecomposition.java


---

Generated on Fri Aug 15 2014 15:11:06 for ESAT by  

 1.8.7
